# Enhanced resolution optoacoustic microscopy using a picosecond high repetition rate Q-switched microchip laser

**DOI:** 10.1117/1.JBO.27.11.110501

**Published:** 2022-11-29

**Authors:** Gianni Nteroli, Giulia Messa, Manoj K. Dasa, Antti Penttinen, Antti Härkönen, Mircea Guina, Adrian Gh. Podoleanu, Stella Koutsikou, Adrian Bradu

**Affiliations:** a University of Kent, Applied Optics Group, Canterbury, United Kingdom; b University of Kent, Medway School of Pharmacy, Chatham, United Kingdom; c Technical University of Denmark, DTU Fotonik, Lyngby, Denmark; d Tampere University, Optoelectronics Research Centre, Physics Unit, Faculty of Engineering and Natural Sciences, Tampere, Finland

**Keywords:** optoacoustic, Q-switched lasers, axial resolution enhancement

## Abstract

Conventional optoacoustic microscopy (OAM) instruments have at their core a nanosecond pulse duration laser. If lasers with a shorter pulse duration are used, broader, higher frequency ultrasound waves are expected to be generated and as a result, the axial resolution of the instrument is improved. Here, we exploit the advantage offered by a picosecond duration pulse laser to enhance the axial resolution of an OAM instrument. In comparison to an instrument equipped with a 2-ns pulse duration laser, an improvement in the axial resolution of 50% is experimentally demonstrated by using excitation pulses of only 85 ps. To illustrate the capability of the instrument to generate high-quality optoacoustic images, *en-face*, *in-vivo* images of the brain of *Xenopus laevis* tadpole are presented with a lateral resolution of 3.8  μm throughout the entire axial imaging range.

## 1 Introduction

Optical imaging techniques used in modern biology, such as confocal, multi-photon, or light-sheet microscopy^1^ require either the use of exogenous probes or genetic manipulations^2^ to achieve the desired optical contrast. Optoacoustic microscopy (OAM) is a hybrid imaging technique employing the absorption of light by intrinsic components of the sample to achieve the desired optical contrast. Exhaustive literature is already available on the use of OAM to image complex biological samples.^3^^,^^4^

In most reports on OAM,^5^^,^^6^ the excitation optical source is a laser delivering pulses of a typical duration of several nanoseconds, used in conjunction with a single-element ultrasound transducer (UT). The values of the pulse energy and duration provided by the source, and the acoustic bandwidth of the transducer, are of paramount importance for achieving high-quality OAM images in terms of signal-to-noise ratio (SNR) and axial resolution. When a very large bandwidth transducer is employed, the axial resolution is limited by the bandwidth of the generated acoustic waves.^7^ So far, the techniques used to enhance the axial resolution have involved either the use of numerical methods requiring long post-processing times or high-frequency UTs.^8^^,^^9^ The number of reports demonstrating improvement in the axial resolution by manipulating the bandwidth of the acoustic waves is limited and typically restricted to situations where the bandwidth is enhanced by reducing the duration of the pulses from hundreds to several nanoseconds.^5^^,^^6^ Using numerical simulations, it has been demonstrated that a 3-ps pulse duration laser is more efficient in generating high-frequency acoustic signals than a 3-ns pulse duration laser, however, no improvement in axial resolution was reported.^10^ To our knowledge, enhancement in axial resolution by reducing the pulse duration below several nanoseconds has not been experimentally demonstrated yet. Here, we show that an OAM imaging instrument, equipped with a Q-switched microchip laser, delivering short pulses of 85 ps at 532 nm, can provide a better axial resolution than when equipped with a supercontinuum optical source delivering excitation pulses of 2 ns, and operating at the same wavelength as the ps laser. The capability of the OAM instrument equipped with the ps-based Q-switched microchip laser to produce high-resolution OAM images is illustrated by images of the brain of the *Xenopus laevis* tadpole.

## 2 Methods and Materials

*In-vivo* imaging was performed on four *Xenopus laevis* tadpoles at developmental stage 37/38 (Nieuwkoop and Faber 1956). *Xenopus laevis* fertilized eggs were purchased from EXRC (Portsmouth, UK) and raised in tap water treated with a commercially available aquarium water conditioner at 20°C. The tadpoles were immobilized in α-bungarotoxin (Invitrogen), placed in saline solution during imaging experiments and remained immobilized during imaging acquisition. All experimental procedures on living tadpoles were approved by the University of Kent Animal Welfare and Ethical Review Body, reference: 0037-SK-17.

**Fig. 1:**
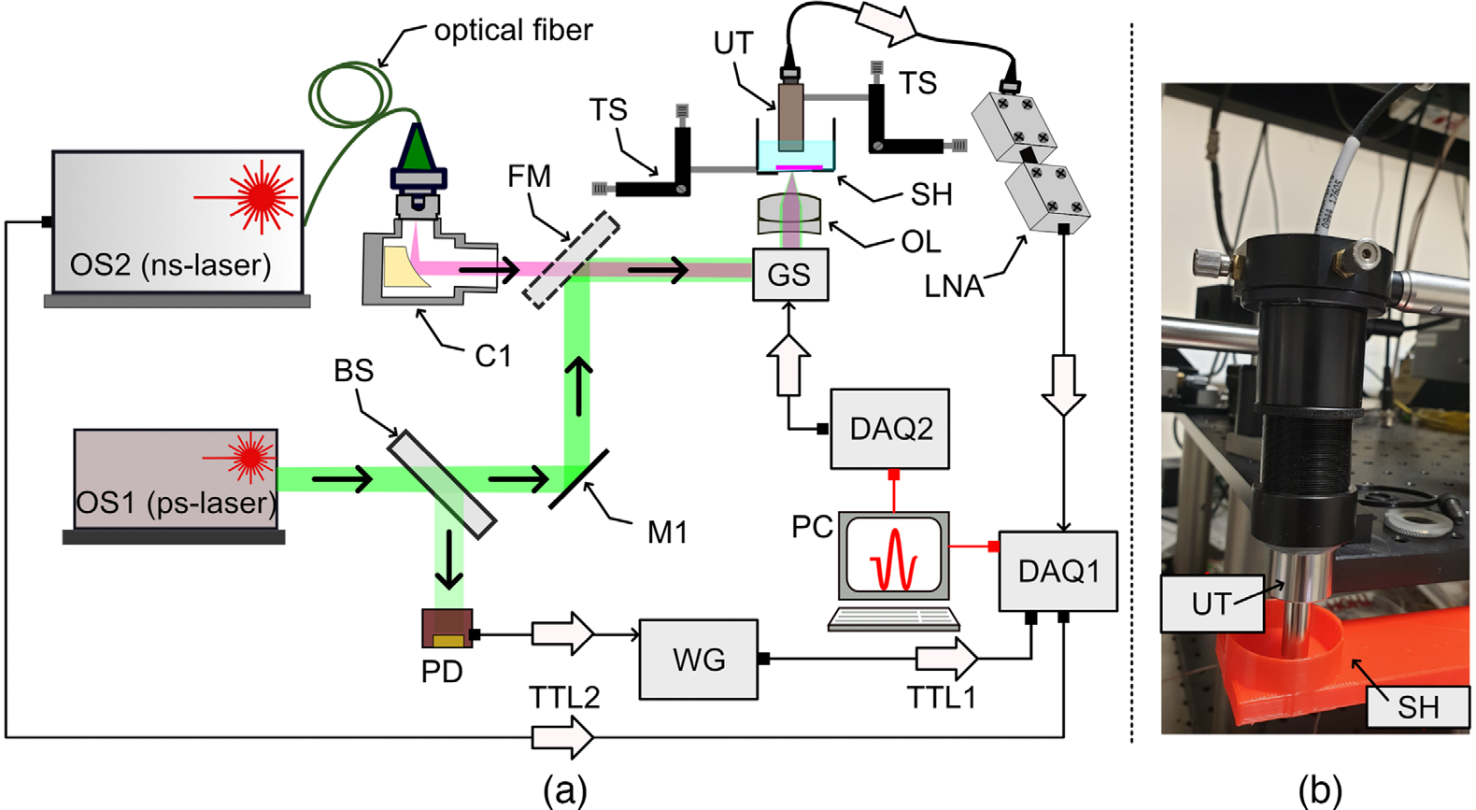
(a) Schematic diagram. OS1: picosecond laser; OS2: supercontinuum optical source; C1: reflective collimator; SH: sample holder; FM: flipping mirror; PD: photodetector; GS: orthogonal galvo-scanners; DAQ1,2: data acquisition cards; LNA: low noise amplifiers; UT: ultrasound transducer; OL: objective lens. TS: translation stage; TTL1,2: TTL signals synchronized with the emission of the pulses. (b) Picture showing the UT and SH.

The schematic diagram of the OAM instrument used in this work is shown in Fig. 1. The optical source (OS1) is a frequency-doubled Q-switched microchip laser emitting at 532 nm (Picophotonics Ltd, Tampere, Finland) capable to generate optical pulses of typically 85 ps and variable output power. OS1 was operated at 60 nJ per pulse and a pulse repetition rate of 50 kHz. The average optical power on the sample was 3 mW. OS2 is a supercontinuum optical source (SuperK Compact, NKT Photonics, Denmark) delivering pulses of 2 ns duration with a repetition rate of 20 kHz. Both OS1 and OS2 delivered the energy per pulse.

The TTL signals (TTL1/2) are used to trigger the digitization of the electrical signal at the output of the low noise amplifiers (LNA) (ZFL-500LN+, Mini Circuits), by a 12-bit data acquisition board, DAQ1 (National Instruments, Model PCI-5124). DAQ1 was operated at a sampling rate of 200 MS/s. The lasers were used sequentially by switching the position of the flipping mirror (FM). The samples were submerged in water to facilitate acoustic coupling. The sample holder (SH) is mounted on a high-resolution 3D translation stage (TS) to position the sample accurately.

The acoustic waves are detected by a high-frequency Polyvinylidene Fluoride ultrasonic transducer (50-MHz central frequency, 53% bandwidth at 6 dB, Model PA1199, Precision Acoustics) placed in contact with the water. The sample lies on an optical window of 0.22-mm thickness, whereas the excitation beam illuminates the sample from below. The electrical signal generated by the transducer is amplified by the two LNAs, before digitization by DAQ1.

As a relatively low central frequency transducer and optical focusing were used to achieve high lateral resolution, our instrument operates in an optical resolution (OR-) OAM regime.

To illustrate the capability of the instrument to produce high-quality images in terms of SNR and spatial resolution, in the picosecond regime, we imaged the brain of *Xenopus laevis* tadpole. The energy per pulse we operated at was 60 nJ, so within ANSI safety standards, which limits the pulse energy to maximum of 1  μJ for OR-OAM instruments.^11^ During imaging, the tadpoles were immobilized and positioned in the SH, (SH in Fig. 1). B-scan images, in the XZ plane, of $400\times 500\;{\mathrm{pixels}}^{2}$ were produced and displayed in real-time at a frame rate of 62.5 Hz (8 ms to capture data and 8 ms to process it). Therefore, XYZ volumes of $400\times 400\times 500\;{\mathrm{pixels}}^{3}$ were generated in 6.4 s. To preserve the lateral resolution throughout the entire axial imaging range, volumetric data were collected for various focusing axial positions of the optical beam inside the tadpole by shifting the SH (in increments of 50  μm) with respect to the objective lens (OL in Fig. 1).

## 3 Results and Discussion

**Fig. 2:**
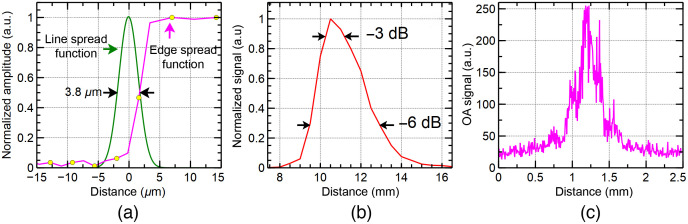
(a) Experimentally measured edge (magenta) and line (green) spread functions. (b) Detected acoustic signal versus axial position (data provided by the manufacturer of the transducer). (c) Lateral FOV, measured by imaging a carbon fiber tape.

Several experiments were conducted to evaluate the capabilities of our OAM instrument. To evaluate the lateral resolution, a sharp edge of a positive USAF target was imaged. Using the image produced, the edge spread function [magenta curve in Fig. 2(a)], and the line spread function [green line in Fig. 2(a)] were calculated. The lateral resolution, defined as the full-width-at-half-maximum (FWHM) of the line spread function, was found to be 3.8   μm, close to the expected theoretical value (3.1   μm using Rayleigh’s criterion^12^). In Figs. 2(b) and 2(c), we show the capability of the instrument in terms of its axial depth of field (DOF), and lateral field of view (FOV), respectively. The axial DOF showed in Fig. 2(b) has been provided by the manufacturer of the transducer, whereas the lateral FOV [Fig. 2(c)] was measured by imaging a carbon fiber tape with OS1. As shown in Fig 2(b), when the OAM signal drops by 3 dB, the axial range is around $z_{\text{max}}=1.5\;\mathrm{mm}$, which represents a sufficiently long axial imaging range to cover a large variety of biological samples, including the *Xenopus laevis* tadpole, which is <1  mm in thickness. As a focused ultrasonic transducer was employed, the OA signal recorded at various lateral positions on the sample shows a maximum in the middle of the image. The lateral FOV, estimated from Fig. 2(c) was $1\times 1\;{\mathrm{mm}}^{2}$.

**Fig. 3:**
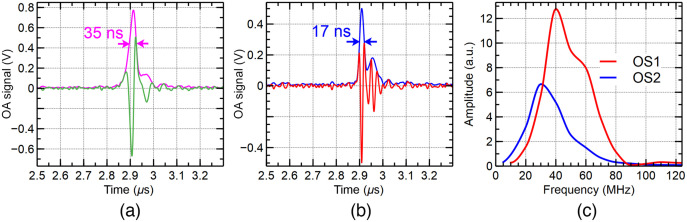
Typical OA signals generated by exciting a carbon fiber with a 2-ns pulse duration [green curve in (a)] and 85-ps pulse duration laser [red curve in (b)]. The envelopes of the two signals are presented in pink and blue, respectively. From the signals presented in (a) and (b), the acoustic spectra generated by using OS1 and OS2 were calculated in (c). By measuring the FWHM of the two spectra, we could infer axial resolutions of 25 and 51  μm, respectively. The fact that the two spectra are not identical in terms of central frequency and bandwidth shows that, the axial resolution is not determined by the bandwidth of the transducer alone.

To experimentally measure the axial resolution, a carbon fiber tape was imaged using both the microchip ps laser, OS1 and the supercontinuum ns source, OS2. Both sources operate at the same central wavelength of 532 nm, however, over different spectral ranges. The ps laser, with an intrinsic bandwidth of a couple of nanometers, delivers sufficient energy per pulse to obtain high-quality images in terms of SNR. To obtain images of similar quality, the light from the supercontinuum source was filtered by a bandpass filter of 25-nm bandwidth. As shown in Fig. 3(a), when pulses of 2-ns duration were employed, the FWHM of the acoustic signal was found to be 35 ns, which, given the speed of sound in soft tissues of ∼1480  m/s, corresponds to an axial resolution of 51  μm. Differently, for an 85-ps pulse duration, the FWHM of the acoustic signal was found to be 17 ns, therefore, an axial resolution of 25  μm [Fig. 3(b)]. This shows that when the bandwidth of the transducer employed is sufficiently wide, the axial resolution can be adjusted by tunning the duration of the pulses. These results were consistent with those obtained in subsequent measurements by imaging different regions of the sample. In Figs. 3(a) and 3(b), only one typical result is presented.

Both picosecond and nanosecond pulse duration optical sources allowed for similar optoacoustic SNRs of 42.9 and 43.8 dB, respectively. In terms of the axial resolution achievable with the two lasers, a 50% enhancement is obtained when using the picosecond pulses in comparison to the nanosecond pulses, when higher frequency acoustic waves are generated as illustrated in Fig. 3(c). The ps-induced optoacoustic signal shows a central frequency at around 41 MHz [Fig. 3(c), red curve], whereas the nanosecond-induced optoacoustic signal shows a central frequency at around 30 MHz [Fig. 3(c), blue curve]. To perform these measurements, the same ultrasonic transducer and sample were employed.

Figure 3(c) shows that the spectrum of the acoustic waves collected using OS1 (red) is larger than the spectrum collected using the OS2 (blue). As a further manifestation of different spectra generated, the centers of the two spectra do not coincide, more acoustic energy is generated by the OS1 closer to the central frequency of the transducer of 50 MHz than when using OS2.

**Fig. 4:**
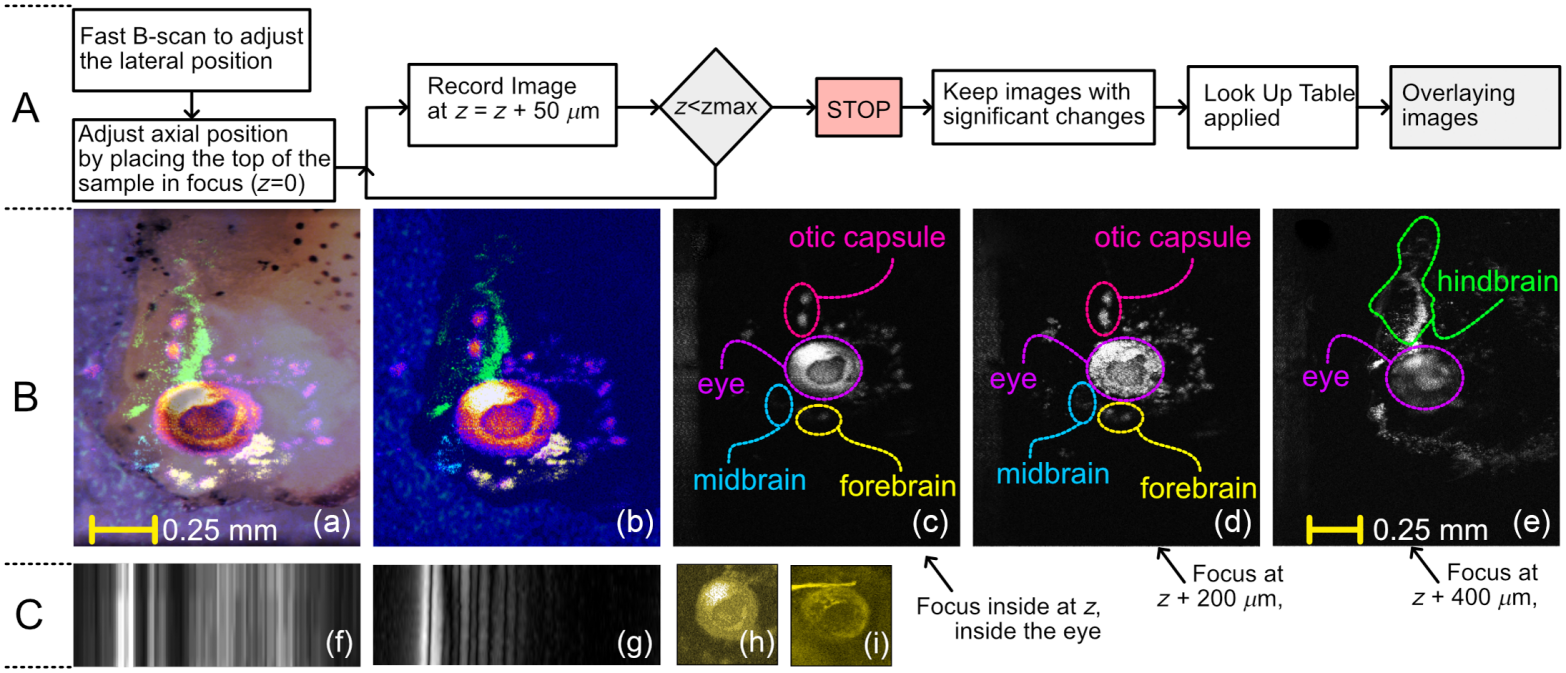
Part A: flowchart describing the imaging protocol and the post-processing steps. Part B: (a) microscope image of the tadpole’s head over which the OAM image showed in (b) is overlapped. (b) Composite *en-face* image obtained by merging images collected at 24 axial positions separated by 50  μm. (c)–(e) Single-plane images showing significant examples of defined brain structures that appear as the focal plane is shifted deeper into the tadpole. The axial separation between (c) and (d), and (d) and (e) is 200  μm. It is noteworthy that (a) has the same lateral size as (b) and (c) has the same lateral size as (d) and (e). Part C: (f) and (g) B-scan images of the carbon tape produced using the ps and the ns lasers, respectively. Axial size (along the horizontal direction): 1.6 mm. Lateral size (vertical direction): 50  μm. (h) and (i), typical *en-face* images of the tadpole’s eye produced using the ps and the ns lasers, respectively. In both cases, the light is focused inside the eye. The artifact in (i) is due to a structural defect of the optical window.

In Fig. 4, *en-face*, high-resolution optoacoustic images at different axial positions inside the tadpole are presented, along with an optical microscopy *en-face* image [Fig. 4(a)]. The *en-face* OAM images were produced by using the maximum intensity projection algorithm. Images obtained at different focusing positions of the optical beam were color-coded (i.e., colors correspond to different focusing positions) and then combined into a composite image such as that depicted in Fig. 4(b). In Figs. 4(c)–4(e), three *en-face* images are presented, which are spaced by 200μm from each other’s axial position. In Fig. 4(c), the focus of the beam is inside the eye, therefore, sharp images of the tadpole’s eye and part of the otic capsule are visible. As a high concentration of melanin is localized in the eye, a high amplitude optoacoustic signal is expected to be generated by the ocular tissue. When the focal plane of the OL is shifted inside the tadpole by 200  μm, the blurred shapes of the midbrain and the forebrain appear [Fig. 4(d)], whereas a sharp image of the eye is not resolved in this focal plane. Moving another 200-μm inward, the hindbrain is displayed, as shown in Fig. 4(e). A flowchart illustrating the procedure employed to record the images and the post-processing steps needed to produce the overlayed images is presented at the top of Fig. 4 (part A).

In Figs. 4(f) and 4(g), we demonstrate B-scan images of the carbon fiber tape produced using the ps and the ns lasers respectively. The axial resolution in Fig. 4(f) is better than in Fig. 4(g). The apparent better depth penetration observable in Fig. 4(f) is possibly due to, (i) the fact that the two images were collected in different experimental conditions (light focused deeper when using the ps laser) and, (ii) not all the wavelengths emitted by the ns laser are probably absorbed by the sample, and therefore, do not contribute to the OAM signal to the extent that the spectrum content of the ps laser is absorbed.

To the best of our knowledge, this level of detail of the tadpole’s brain shown in Fig. 4 has not been demonstrated yet by any other research group using an OAM instrument equipped with a ps pulse duration laser.

## 4 Conclusion

In this letter, we demonstrated that the axial resolution of any OAM instrument can be improved by narrowing the pulse duration of the excitation laser, therefore high-axial resolution images can be produced. More precisely, we experimentally proved that by using a Q-switched microchip laser delivering 85-ps pulses, the axial resolution is 50% better than when employing a 2-ns pulse duration laser. It is expected that by using narrower laser pulses, the axial resolution to be improved even further. By simulation, a 3-ps pulse duration laser has been shown^10^ to generate frequencies well above 270 MHz. In principle, if the conditions to produce the photo-acoustic effect are fulfilled, extremely low pulse duration lasers can be used to develop high-axial resolution instruments. Extreme short pulses however for similar energies, may exhibit such high peak pulse power that nonlinear optical effects may limit further reduction of the pulse duration. Compared to other types of optical sources used in OAM, such as frequency-converted ns Q-switched lasers^13^^,^^14^ or supercontinuum, fiber systems^15^^,^^16^ the microchip laser employed, although at the moment is only capable to operate at a single wavelength, offers other advantageous features, such as a higher pulse repetition rate than standard Q-switched systems, and/or higher pulse energy compared to mode-locked systems. Moreover, the laser has a small footprint ($15\times 15\times 12\;{\mathrm{cm}}^{3}$), a rather simple architecture, and low cost, which makes it advantageous also compared to solutions based on amplified gain-switched lasers. Although the ps laser employed here only operates at 532 nm, we must point out that 532 nm is a very popular wavelength used for opto-acoustic imaging as light at this wavelength is absorbed by a plethora of chromophores present in biological samples. Finally, a quite high repetition rate suggests that the chip laser employed here can be a highly sought-after optical source for OAM. To take full advantage of the enhancement in axial resolution, further investigations are needed as soon as faster OA transducers become available, and more shorter laser pulse technologies are developed. As of now, we need to mention two limitations of the instrument we developed due to the repetitive nature of the experiments conducted. First, to achieve constant high lateral resolution along the axial direction, and therefore be able to differentiate the anatomical brain structures of the tadpole, repetitive imaging at 50  μm increments was required and second, although the B-scan images were produced in real-time, the combined image shown in Fig. 4 required post-processing. To overcome these limitations, higher pulse repetition rates must be used in an instrument equipped with a fast-focusing capability (such as the use of a liquid lens, instead of the manual adjustment we performed) and harness the computing power of the graphics cards to improve the post-processing time.
